# Effect of phosphorus stress on *Microcystis aeruginosa* growth and phosphorus uptake

**DOI:** 10.1371/journal.pone.0174349

**Published:** 2017-03-22

**Authors:** Sajeela Ghaffar, R. Jan Stevenson, Zahiruddin Khan

**Affiliations:** 1 Institute of Environmental Sciences and Engineering, School of Civil and Environmental Engineering (IESE-SCEE), National University of Sciences and Technology (NUST), Sector H-12 Campus, Islamabad, Pakistan; 2 Center for Water Sciences & Department of Integrative Biology, Michigan State University, East Lansing, Michigan United States of America; 3 Institute of Environmental Sciences and Engineering, School of Civil and Environmental Engineering (IESE-SCEE), National University of Sciences and Technology (NUST), Sector H-12 Campus, Islamabad, Pakistan; INRA, FRANCE

## Abstract

This study was designed to advance understanding of phosphorus regulation of *Microcystis aeruginosa* growth, phosphorus uptake and storage in changing phosphorus (P) conditions as would occur in lakes. We hypothesized that *Microcystis* growth and nutrient uptake would fit classic models by Monod, Droop, and Michaelis-Menten in these changing conditions. *Microcystis* grown in luxury nutrient concentrations was transferred to treatments with phosphorus concentrations ranging from 0–256 μg P∙L^-1^ and luxury nitrogen. Dissolved phosphorus concentration, cell phosphorus quota, P uptake rate and cell densities were measured at day 3 and 6. Results showed little relationship to predicted models. *Microcystis* growth was asymptotically related to P treatment from day 0–3, fitting Monod model well, but negatively related to P treatment and cell quota from day 3–6. From day 0–3, cell quota was negatively related to P treatments at <2 μg∙L^-1^, but increased slightly at higher P. Cell quota decreased greatly in low P treatments from day 3–6, which may have enabled high growths in low P treatments. P uptake was positively and linearly related to P treatment during both periods. Negative uptake rates and increases in measured culture phosphorus concentrations to 5 μg∙L^-1^ in the lowest P treatments indicated P leaked from cells into culture medium. This leakage during early stages of the experiment may have been sufficient to stimulate metabolism and use of intracellular P stores in low P treatments for rapid growth. Our study shows P regulation of *Microcystis* growth can be complex as a result of changing P concentrations, and this complexity may be important for modeling *Microcystis* for nutrient and ecosystem management.

## Introduction

Freshwater harmful algal blooms, often dominated by cyanobacteria, are speculated to increase with climate change and are expected to decrease the potable and recreational uses of water [1–2]. *Microcystis aeruginosa* (referred to as “*Microcystis*” in this paper for convenience) is a common constituent of harmful cyanobacterial blooms [3–5]. Many strains of *Microcystis* produce microcystin, a hepatotoxin, which enters the food chain and disrupts ecological balance [6] and is a human health hazard [7]. Thus, *Microcystis* has been a big problem and has been studied worldwide in freshwater ecosystems [8–11].

*Microcystis* has a number of adaptive features that make it a successful competitor such as gas vesicles [12], luxury consumption of phosphorus and storage as polyphosphate granules [13], tolerance to high irradiance [14], and large colonies with mucilage that reduce grazing [5]. Furthermore, they overwinter in benthic sediments and the water column [15].

Understanding the growth kinetics of *Microcystis* in relationship to the nutrient concentrations that lead to its blooms is vital for managing them [16]. Field surveys and experiments provide two sources of information for resource managers showing phosphorus is the most limiting nutrient in freshwater bodies [17–18] and relationships between cyanobacterial abundance and phosphorus concentrations in lakes [10, 19]. Process-based models are another source of information needed by resource managers, and relationships between cell growth rates and nutrient uptake rates are key elements in these models. Three models are commonly used to characterize cell growth and nutrient uptake rates: Michaelis-Menten nutrient uptake model [20], Monod’s extracellular nutrient and cell growth model [21], and Droop’s intracellular nutrient and growth model [22]. Parameters from Monod models are the foundation of process-based models of algal blooms, e.g. BASINS, which provides resource managers with important complements to experiments and empirically-derived statistical models when conducting causal analyses and predicting results of management [23–24].

Many studies have measured *Microcystis* response to phosphorus concentrations in experiments, but they usually lack sufficient information to parameterize nutrient uptake and growth models. Early studies by Gotham and Rhee [25] and Olsen et al. [26] do not have sufficient range and distribution in phosphorus concentration as well as sufficient sample size to enable accurate characterization of half saturation constants and maximum uptake and growth rates. Relationships between phosphorus concentration and P uptake rates and cell growth rates are not described or illustrated in ways that enable evaluation of sample size, range, and distribution in more recent studies by Ou et al. [27] and by Chen et al. [28] (as cited with [27]). Therefore, despite the significance of *Microcystis*, few studies have characterized its growth kinetics by simultaneously measuring phosphorus uptake rates, phosphorus concentrations in cells, and cell growth rates with high replication and treatment numbers. All these measurements and high sample numbers are needed to statistically distinguish details in relationships between phosphorus conditions of water and cells and rates of cell growth and nutrient uptake. In addition, little has been done to understand response of *Microcystis* growth kinetics in experiments when phosphorus conditions change, which is common in lakes. Thorough characterization of growth kinetics and phosphorus condition will be particularly important to evaluate *Microcystis* response to changing phosphorus concentrations.

This study aims to advance understanding of *Microcystis* growth and phosphorus uptake kinetics in changing phosphorus concentrations by employing and evaluating multiple modeling approaches. *Microcystis* growth and phosphorus uptake rates were measured in a range of phosphorus concentrations in the laboratory immediately after growing them in high phosphorus concentrations. We measured *Microcystis* responses to phosphorus manipulations during two successive three day periods to gain more information about responses to changing phosphorus conditions. We tested whether the classic Michaelis-Menten, Monod, and Droop models explained *Microcystis* growth and compared them to linear and polynomial models to quantify the relationships between growth rates, phosphorus uptake kinetics, and phosphorus concentrations inside and outside cells.

## Methodology

### Experiment

The *Microcystis* strain was obtained from Dr. Orlando Sarnelle’s laboratory in the Department of Fisheries and Wildlife at Michigan State University. It was isolated from a hypereutrophic lake in the Michigan State University Inland Lakes Research Area about 1.5 years before our experiment. The seed cultures of *Microcystis* were grown in half strength WC medium for one week on a 12h/12h light/dark cycle and a light intensity of 20 μmol·m^-2^·sec^-1^. Subcultures were seeded weekly from this stock into fresh half-strength WC medium to grow sufficient *Microcystis* for the experiment.

Forty-four glass Erlenmeyer flasks of 150mL capacity were acid washed, dried, and then used for the experiment. The amount of subculture to be inoculated was selected to produce a cell density that could be counted microscopically without concentration and was in the range of densities found in lakes, which was established as 3x10^4^ cells per mL. The subculture for seeding was filtered under very low suction and then carefully washed with half strength WC medium having no phosphorus to minimize carrying-over of surface adsorbed phosphorus into the flasks. This approach of washing and transfer to experimental flasks has also been used by Tsukada et al. [16], and we observed no ill effects of washing during the first 3 days of our experiment when *Microcystis* growth rates increased asymptotically with phosphorus treatment, as expected. All flasks had 100 mL of culture medium and were then seeded with the fixed amount of subculture. Three flasks were inoculated and immediately sampled on day 0 to confirm the number of cells inoculated.

We used batch culture methods for our experiment, i.e. the culture was not maintained at a specific growth stage with constant addition and removal of culture medium and cells [29]. The rationale of using a batch culture was derived from the fact that a natural ecosystem is not steady-state. Nutrient concentrations vary with weather-related changes in nutrient loading, after which no resupply of nutrients to the water column can be expected. Thus *Microcystis* habitats are more like a batch experiment than continuous cultures that can reach steady state.

The experiment was conducted at 28±1°C temperature, 30 μmol·m^-2^·sec^-1^ light intensity and 12h/12h light/dark cycle. Ten (10) phosphorus treatments with WC medium were administered with four replicates for each treatment. A set of four control flasks was run that had no phosphorus added to the medium. The other ten treatments were 0.5, 1, 2, 4, 8, 16, 32, 64, 128 and 256 μg PO_4_-P L^-1^. We selected this exponential increase in phosphorus concentration for treatments so half of the treatments had very low concentrations to increase chances of accurate characterizations of uptake and growth rates in ranges of nutrient concentrations that we expect the greatest changes; and half of the treatments had relatively high nutrients in which uptake and growth rate were expected to be near or at maximum rates. The experiment was run for 6 days with samples drawn from each replicate on day 3 and day 6 to estimate biomass (estimated as number of cells per mL), dissolved phosphorus (water column concentration), and intracellular phosphorus (cell phosphorus quota). The only time cultures were shaken was when samples were collected on day 3 and day 6. No settling of *Microcystis* was observed in the flasks.

A total of 35 mL was drawn from each flask with 25 mL used for phosphorus analysis and 10 mL used for cell counts. 35 mL of culture medium with treatment phosphorus concentrations were added to each flask to replace the 35 mL withdrawn. The 25 mL phosphorus subsample was filtered through GF/F filters with 0.7 μm pore size. The filtrate was processed for dissolved phosphorus analysis and frozen in acid rinsed polyethylene bottles until analyzed for dissolved phosphorus. Particulate matter and GF/F filters were wrapped in aluminum foil, frozen, and stored for organic phosphorus analysis to estimate cell quota. Samples for cell counts were preserved in glass vials with glutaraldehyde until analysis. Glutaraldehyde was added with micropipette to a final concentration of 1% v/v.

### Sample analysis

In preparation for analysis of phosphorus concentrations in filtrate and *Microcystis* cells, water and filter samples were thawed. Particles trapped on GF/F were pre-combusted at 500°C in a muffle furnace. The alkaline persulfate method was employed to digest the organic phosphorus in cells for cell phosphorus quota measurement [30]. Potassium persulfate oxidizing solution was added to samples and autoclaved for forty-five minutes. PO_4_-P was then measured by the ascorbic acid method using a spectrophotometer ([30]; Spectronic Genesys 10, USA).

Cells of *Microcystis* were counted in a Palmer Maloney chamber that holds 0.1 mL sample, under 400X magnification with a Leica DMLB research-quality microscope. No less than 40 fields of view were counted for each sample. Cells per milliliter were calculated with the formula in APHA Standard Methods [30].

### Data analysis

Cell growth rates and phosphorus uptake rates were calculated for two periods: day 0 to day 3 and day 3 to day 6. Cell growth rates (d^-1^) were calculated by dividing the difference in natural-log transformed cell densities in cultures between successive sampling dates by the number of days between successive sampling dates. Calculations for growth rates during the day 3 to day 6 period were corrected for withdrawing 35% of the cells during sampling on day 3. Day 3 cell densities when cultures were sampled were multiplied by 0.65 to calculate the cell densities in cultures after sampling and addition of 35 mL of culture medium. Phosphorus uptake rates (pg PO_4_-P∙cell^-1^∙d^-1^) were calculated by dividing the difference in mass of PO_4_–P between successive sampling dates by the average number of cells in the culture on the two dates used in the calculation. The removal of 35 mL of culture on day 3 and replacement with medium with appropriate phosphorus concentrations was also accounted for in estimates of the mass of PO_4_-P in cultures when calculating phosphorus uptake rates for the period from day 3 to day 6.

Coefficients of the Monod and Droop growth models were used to characterize the relationships between cell growth rates and P concentration. Monod’s model is mathematically analogous to Michaelis-Menten kinetics. It explains growth rate of cells (μ) as a function of the concentration of a single extracellular nutrient [21]:

$$µ={µ}_{max}.\frac{S}{K_{s}+S}$$

Here μ_max_ is the maximal growth rate and K_s_ is the half saturation constant, which is defined as the nutrient concentration in the water column (S) when growth rate (μ) is half that of maximum growth rate. It assumes that growth rates increase rapidly at low nutrient concentrations and then saturate at higher concentrations, such that organisms achieve a maximum growth rate and growth rate becomes independent of substrate concentration [31], thereby fulfilling the kinetic principle proposed by Penfold and Norris [32]. The curve plotted on Monod’s growth model is a rectangular hyperbola. Monod’s growth model has also been criticized for deviations of growth rate at low nutrient concentrations, predicting higher than actual values [31].

Droop’s growth model [22] varies from Monod’s regarding effective nutrient concentration for cell growth. It relates growth rate (μ) to intracellular nutrient concentration, which is called the cell quota (Q); hence the model is referred to as the “cell quota model”:

$$µ={µ´}_{max}.(1-\frac{K_{Q}}{Q})$$

Here μ´_max_ is the growth rate that can be achieved at saturating cell quota (i.e. Q_max_). Theoretically, it is higher than Monod’s μ_max_, [33] but practically, both are identical [34]. Q_o_ is the minimum cell quota below which growth rates are less than or equal to zero, or the “subsistence quota” as called by Droop [22]. The limitation to the Droop model is a difficulty in measuring the intracellular nutrient concentration in a field water sample, because it is a mixed culture containing multiple species of algae, protozoa, zooplankton, and detritus [34]. Also, it is harder to comprehend the biological interpretation of cell quota compared to dissolved nutrient concentration [35]. The lack of a strong mechanistic foundation is also cited as its weakness [36–37]. Coefficients for both were calculated using nonlinear least squares regression function in R ver 3.0.1 [38].

Parameters of the Michaelis-Menten nutrient uptake model was used to characterize the relationship between PO_4_ uptake rate (P) and concentration (S), where:

$$P=P_{max}.\frac{S}{K_{p}+S}$$

P_max_ is the maximal uptake rate and K_p_ is the half saturation coefficient for uptake rate. S is the substrate concentration at day 0, i.e. the planned treatment concentration.

Coefficients for the Monod, Droop, and Michaelis-Menten models were calculated using non-linear least squares regression with R ver 3.0.1 [38]. Goodness of fit of models was evaluated using R^2^ values [39–40], standard errors, and residual sum of squared (RSS). Also, systematic changes in residuals along the phosphorus gradient were used to evaluate goodness of model fit. Patterns in residuals along the phosphorus gradient would indicate that these models did not explain unknown aspects of phosphorus uptake and growth kinetics in cells, as explained by Wisniak and Polishuk [41]. Nonlinear regression was used to evaluate systematic changes in residuals along the phosphorus gradient created by experimental treatments using R ver 3.0.1 [38]. To even the distribution (and statistical influence) of phosphorus treatment level along the phosphorus gradient, various transformations of phosphorus concentration were evaluated. We chose log transformation of the phosphorus treatment concentration in residual analyses for Monod and Michaelis-Menten models. For Droop model, untransformed values of cell quota were used to analyze residuals. The residual analyses are not presented in the paper, but are available upon request.

When Monod, Droop, and Michaelis-Menten models did not fit relationships between *Microcystis* growth kinetics and phosphorus concentrations, we used a curve fitting protocol to determine whether other relationships existed with phosphorus concentrations. Linear regression, polynomial regression, and piecewise linear regression using R ver 3.0.1 were used in this protocol. Goodness of model fit and selection of the best models were again determined with R^2^ values, standard errors, and residual sum of squared (RSS). For selection as the best models, nonlinear models derived from polynomial regression or piecewise linear regression had to have goodness of fit statistics about 10% better than linear models.

## Results

**Fig 1:**
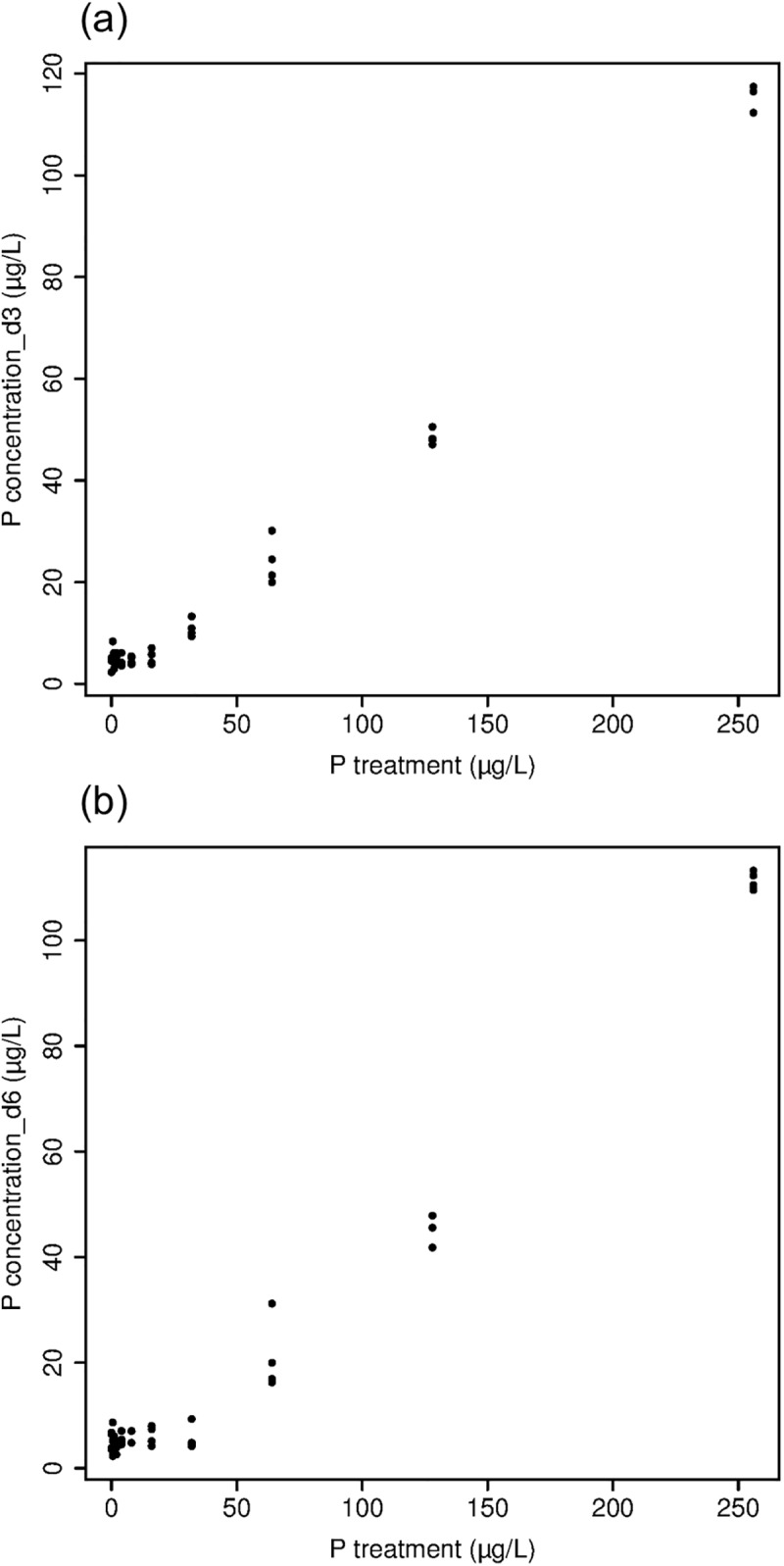
**Phosphorus concentrations in culture media in each of the phosphorus treatments on day 3 (a) and on day 6 (b)**.

Variability in phosphorus concentrations within P treatment was relatively low and clearly showed effects of P treatment (Fig 1), with potential leakage of phosphorus in low P treatments and phosphorus uptake in high P treatments. Phosphorus concentrations in cultures on day 3 averaged about 5 μg L^-1^ and varied little among P treatments ranging from 0 to 16. Phosphorus concentrations in cultures on day 3 increased steadily from 5 to 115 μg L^-1^ with P treatments from 16 to 256. Phosphorus concentrations in cultures on day 6 differed little from day 3, averaging 5 μg L^-1^ in P treatments from 0 to 32 and increasing with successively higher P treatment to 111 μg L^-1^ in the 256 P treatment.

**Fig 2:**
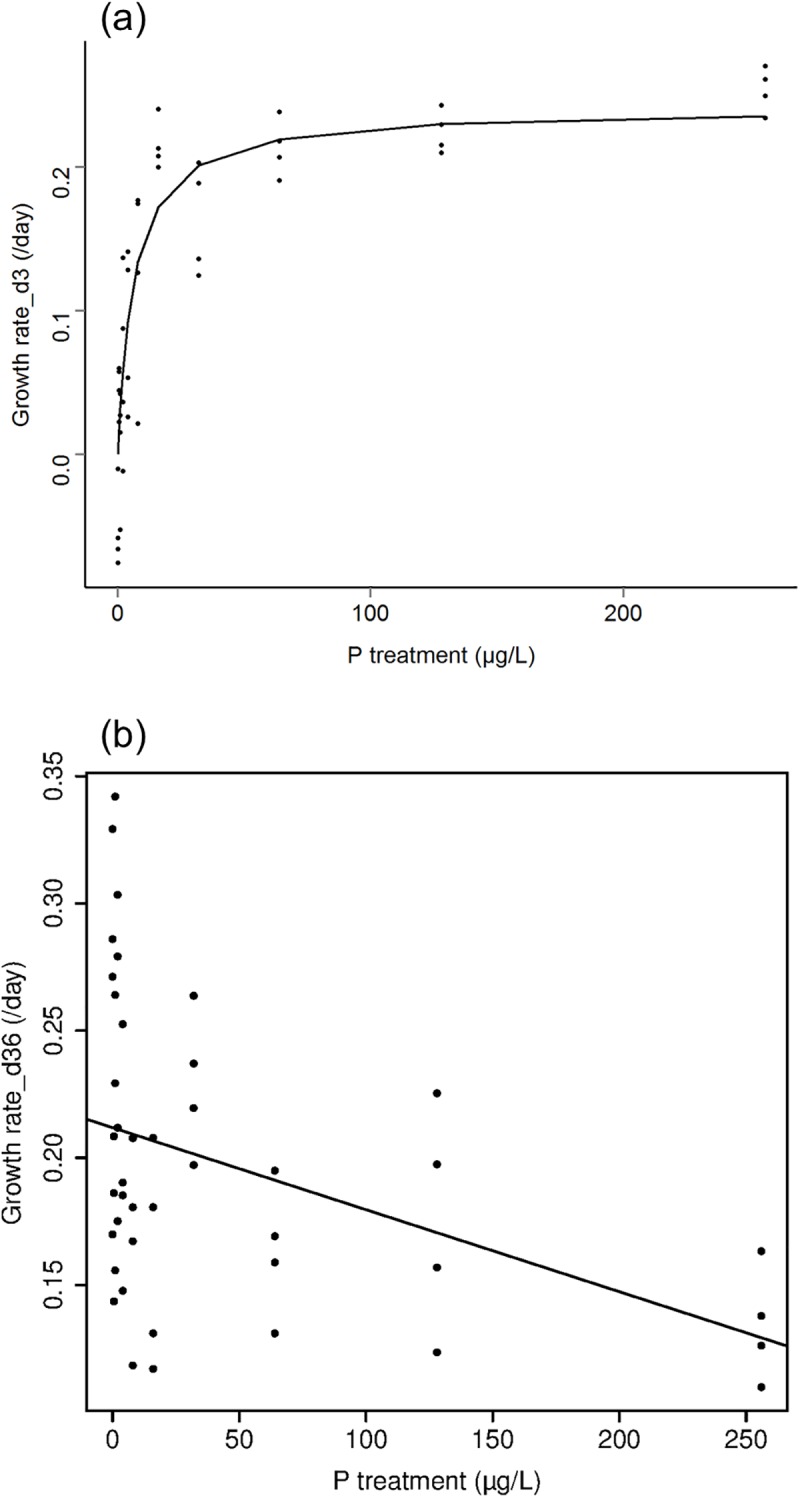
**Microcystis growth rates in each of the phosphorus treatments for the day 0–3 period (a) and day 3–6 period (b).** The asymptotic line in Fig 3A is the best fit to the Monod model. The linear line in Fig 2b is the best model for the day 3–6 relationship.

Cell densities in cultures increased asymptotically (rectangular hyperbola) with P treatment on day 3 and day 6. They ranged from 25,000 to 65,000 cells mL^-1^ on day 3 and from 35,000 to 70,000 cells mL^-1^ on day 6. The growth rates of *Microcystis* that generated these cell density patterns from day 0 to day 3 ranged from -0.08 day^-1^ to 0.27 day^-1^ and increased asymptotically with phosphorus treatment level (Fig 2A). Growth rates increased most in the P treatment range of 0 to 16 μg L^-1^ and increased little at higher treatment levels. The Monod model estimated with non-linear regression and phosphorus concentration measured in cultures had a μ_max_ of 0.27, a K_s_ of 10.7, and a residual standard error of 0.055.

From day 3 to 6, *Microcystis* growth rates ranged from 0.11 to 0.34 day^-1^, were higher overall than from day 0 to 3, and were negatively related to P treatment concentration by linear regression (p = 0.004, R^2^ = 0.16, Fig 2B, Table 1). The Monod model did not fit the growth-P treatment relationship. The linear model relating growth rate to P treatment had an intercept of 0.212 and slope of -0.0003. Growth rates of *Microcystis* in low P treatments were much higher during the day 3–6 period than the day 0–3 period, but in high P treatments growth rates were somewhat lower during the day 3–6 than 0–3 period.

**Table 1:** Summary statistics of various relationships drawn to understand Microcystis physiology.

Relationship	Regression type	Residual sum of squares (RSS)	R^2^	Slope	Intercept	Residual standard error (RSE)^1^	p value
Growth rate_03 = f(P treatment)	Nonlinear	0.083	0.9	—	—	0.045	—
Growth rate_36 = f(P treatment)	Linear	—	0.15	-0.0003	0.212	0.053	0.004
Growth rate_03 = f(Cell quota_d3)	Linear	—	0.34	-0.0003	0.34	0.083	<0.001
Growth rate_36 = f(Cell quota_d6)	Linear	—	0.15	-0.0001	0.25	0.053	0.005
Cell quota_d3 = f(P treatment)	Linear	—	-0.02	-0.0054	637	183.2	0.988
	Piecewise	—	0.415			138.4	<0.001
Cell quota_d6 = f(P treatment)	Linear	—	0.59	1.77	324.4	113.2	<0.001
	Piecewise	—	0.65			104.9	<0.001
Uptake rate_03 = f(P treatment)	Linear	—	0.98	0.0042	-0.011	0.049	<0.001
Uptake rate_06 = f(P treatment)	Linear	—	0.92	0.0014	-0.003	0.031	<0.001

^1^ df = 42.

**Fig 3:**
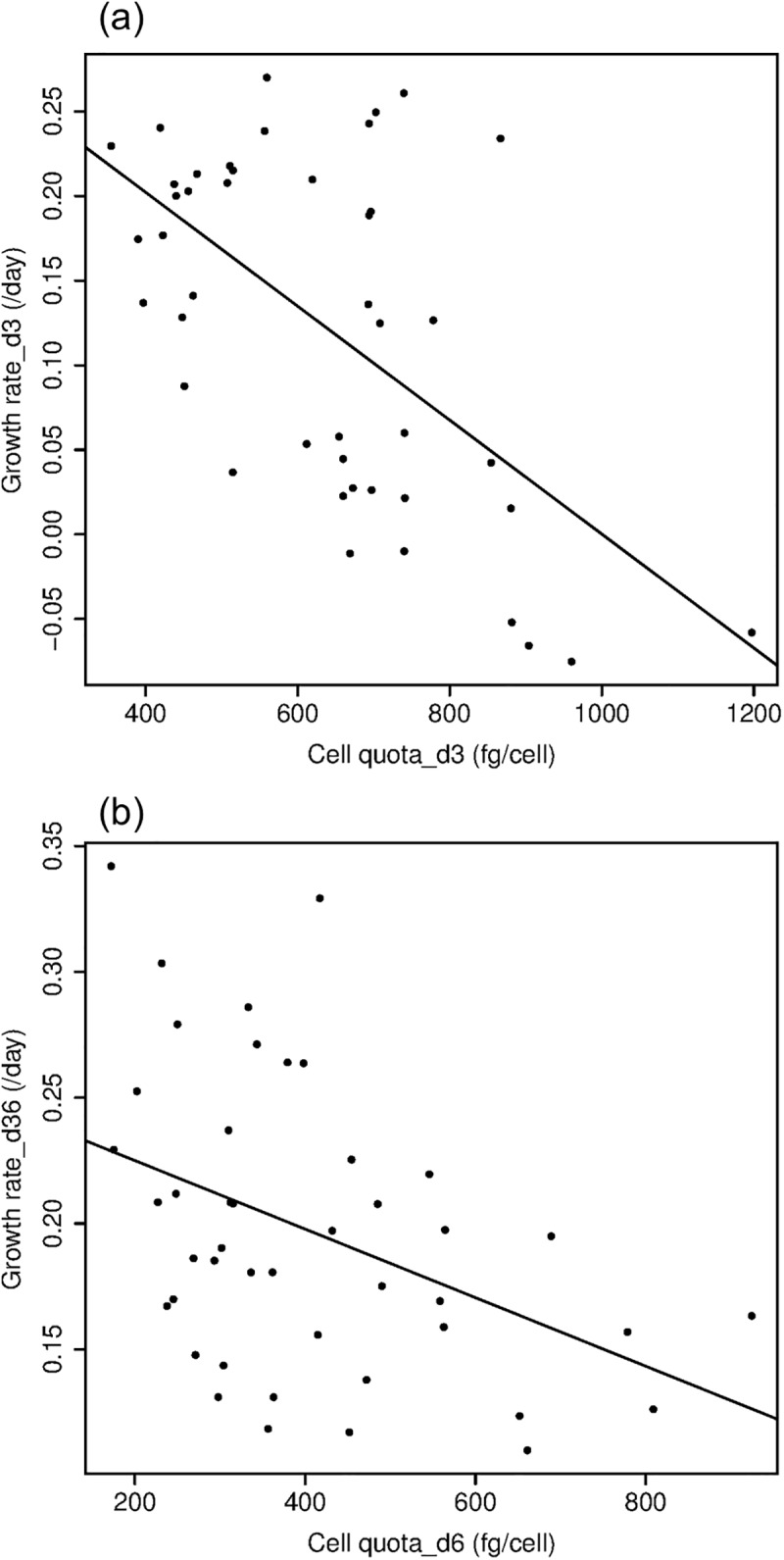
Microcystis growth rates related to cell quota. Growth rates for day 0–3 versus cell quota on day 3 are plotted in Fig 3a. Growth rates for day 3–6 are related to cell quota on day 6 in Fig 3b. The linear models in both figures were the best model and fit for these relationships. (Note the difference in Y-axis.)

Droop’s asymptotic model for the relationship between growth rates and cell quota was not observed during our experiment. Growth rates were negatively related to cell quota by linear regression from day 0 to 3 and from day 3 to 6 (Fig 3, Table 1). The negative relationship between growth rates and cell P quota was greater from day 0 to 3 (slope = -0.003, R^2^ = 0.34, p<0.001) than from day 3 to 6 (slope = -0.001, R^2^ = 0.15, p = 0.005).

**Fig 4:**
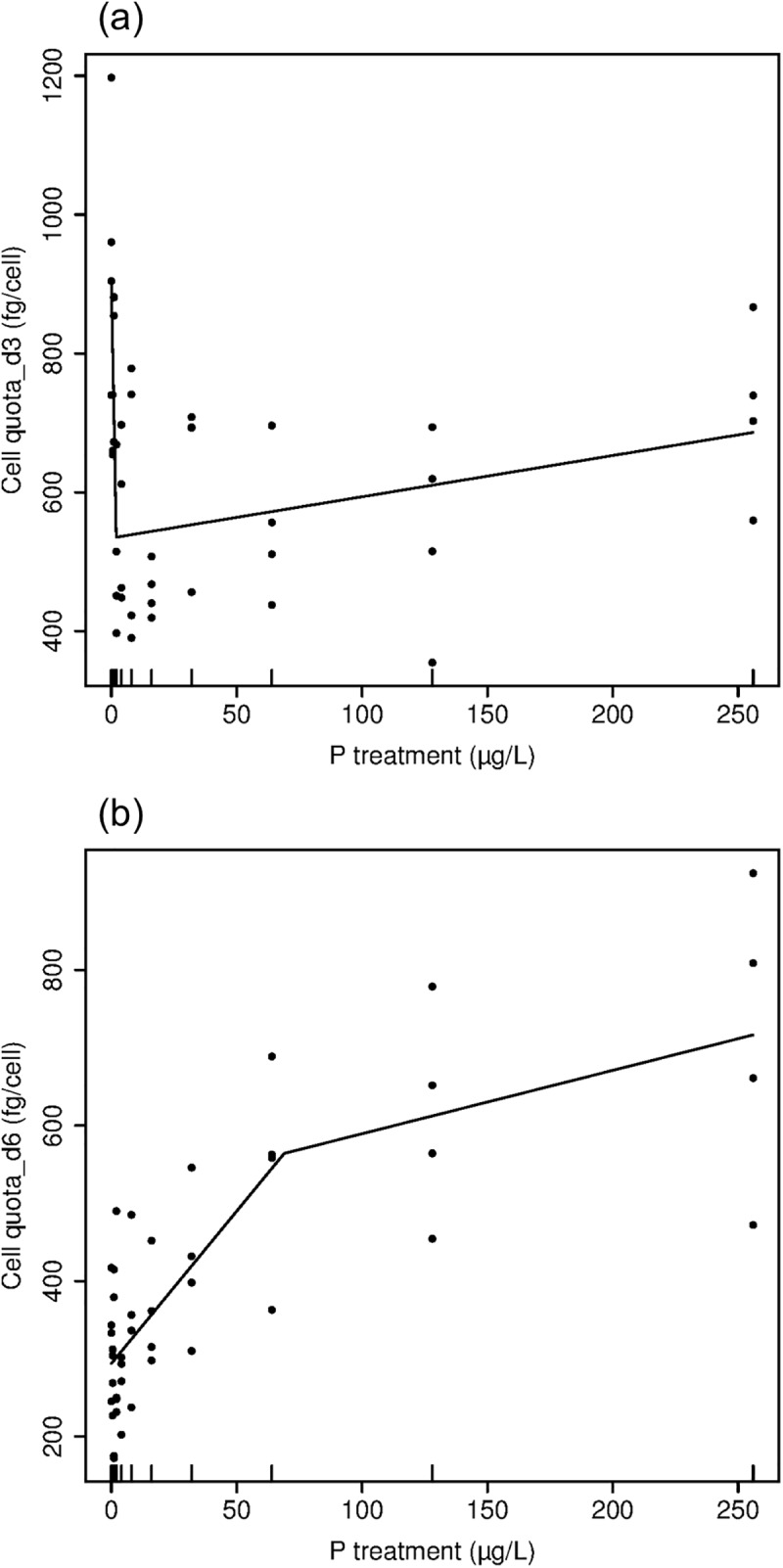
The relationships between cell quota and phosphorus treatment. Fig 4a and 4b show the results of piecewise linear regression for cell quota on day 3 and day 6, respectively. (Note the difference in Y-axis.)

Temporal and treatment-related changes in cell quota indicated complex internal P dynamics. Cell quota on day 3 tended to decrease with increasing P treatments ranging from 0 to 16 and then increased with successively higher P treatment (piecewise linear regression, P<0.001, breakpoint = 1.95 μg/L, Fig 4A). Cell quota decreased from an average of 950 fg cell^-1^ on day 3 in the 0 P treatment to around 500 fg cell ^-1^ in the P treatments ranging from 2–16. In high P treatments on day 3 cell quota increased from 300 fg cell^-1^ in low P treatments to an average of 717 fg cell^-1^ in the 256 P treatment. Cell quota in low P treatments (0, 0.5, and 1) decreased from day 3 to day 6 by approximately 600 fg cell^-1^. Cell quota on day 6 increased with P treatment with a somewhat higher rate of increase in low-intermediate P treatments than in in the intermediate-high P treatment range (piecewise linear regression, p<0.001, breakpoint = 68.8 μg/L, Fig 4B). The average cell quota in the 256 P treatment on day 6 was 717 fg cell^-1^, which is exactly the same as on day 3 in the 256 P treatment.

**Fig 5:**
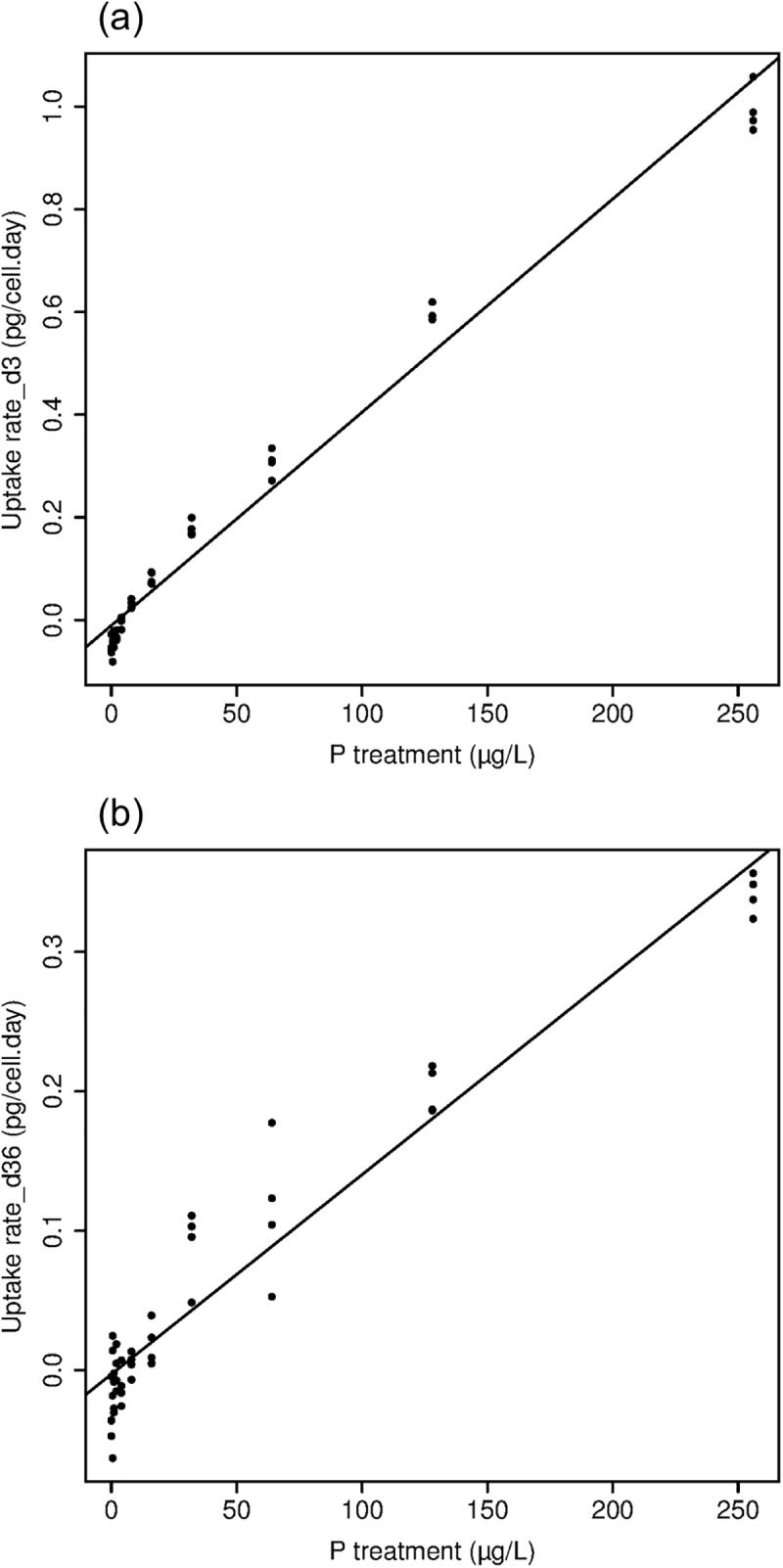
**The relationship between phosphorus uptake rates and phosphorus treatment for day 0–3 (a) and for day 3–6 (b).** Linear models which were the best fit to the relationships. (Note the difference in Y-axis.)

The relationship between phosphorus uptake rates was not fit well by the Michaelis-Menten model from day 0 to 3 or from day 3 to 6. Uptake rate was positively and linearly related to P treatment during both periods (Fig 5). Phosphorus uptake rate ranged from -0.08 pg∙cell^-1^∙day^-1^ to 1.06 pg∙cell^-1^∙day^-1^ from day 0 to 3, when the linear model (p = <0.001) had an R^2^ value of 0.98 with a slope of 0.0041 and intercept of -0.011.

For the next 3 days of experiment, the uptake rate ranged from -0.63 pg∙cell^-1^∙day^-1^ to 0.36 pg∙cell^-1^∙day^-1^, when the linear model (p = <0.001) had an R^2^ value of 0.92 with a slope of 0.0014 and intercept of -0.003. Thus uptake rates decreased and the rate of increase in uptake with P treatment decreased from the day 0–3 to day 3–6 period.

## Discussion

We aimed to understand the growth and uptake responses of *Microcystis* to a gradient of phosphorus concentrations during a short, 6-day period after a period of growth in saturating nutrient concentrations. The range of phosphorus concentrations in the experiment were sufficient to reduce phosphorus uptake and growth rates of *Microcystis* to near zero or below in low phosphorus concentrations and to saturate growth rates in high phosphorus concentrations. Uptake rates were not saturated in high P treatments. We expected growth and uptake physiology of *Microcystis* to fit Monod, Droop, and Michaelis-Menten model predictions along the phosphorus gradient, but cell growth and phosphorus uptake rates did not fit these models consistently over the 6-day period. Our results are likely due to response of cells to changing phosphorus at the start of the experiment as well as over the course of the 6-day period of the experiment, and they can be explained by the basic physiological theory underpinning the Monod, Droop, and Michaelis-Menten model plus cell phosphorus leakage and intracellular P hoarding in very low P treatments. We will discuss our results and this explanation in the following paragraphs.

The Monod model had a good fit for the relationship between growth rates and phosphorus treatment concentrations during the day 0 to day 3 period. The kinetic parameters from our study during this period were lower than those reported in other studies [28] and [42] as described in [27]. Chen et al. [28] reported K_s_ and μ_max_ values of 0.412 μmol∙L^-1^ and 1.98 day^-1^ respectively. Ou et al. [27] reported a K_s_ of 0.0352 μmol∙L^-1^ and μ_max_ of 0.493 day^-1^. Qu and Liu [42] estimated coefficient values of K_s_ as 0.548 μmol∙L^-1^ and μmax as 1.143 day^-1^. The lower μ_max_ values in our study may have been caused by lower light intensity in our study, 30 μmol∙m^-2^∙s^-1^, as compared to 60 μmol∙m^-2^∙s^-1^ in Ou et al. [27]. Genetic differences in strains of *Microcystis* studied may also have affected physiological performance. Our growth rates also may be related to using batch versus continuous culture methods, because actual nutrient concentrations in batch culture are lower than initial concentrations; whereas the initial nutrient concentrations in continuous culture are maintained throughout the experiment with a continuous supply of nutrients creating a steady-state growth condition.

The relationship between *Microcystis* growth rate and P treatment concentration changed greatly from the day 0–3 to day 3–6 period. From day 3–6, *Microcystis* growth rate was negatively related to P treatment concentration. Most of the change between successive culture periods was due to a great increase in growth rates in low nutrient concentrations. Growth rates of *Microcystis* in low P treatments from day 3–6 were as high or higher than the μ_max_ estimated in the Monod model for the day 0–3 period. We hypothesize this surge in growth rates in low P treatments is complexly related to leakage of nutrients from cells into cultures and to a non-linear relationship between utilization of intracellular phosphorus stores and external phosphorus concentrations.

Intracellular nutrient ratios and their concentrations reflect their relative and absolute availability for cell growth. If uptake and changes in cell quota during culture were affecting growth rates, then we would expect to see a positive relationship between cell quota and growth rates as predicted by Droop [22]. Instead, we observed negative relationships between growth rates and cell quota. However, this is a steady-state representation of a non-steady state condition; we did see positive responses in cell growth rates in relation to temporal decreases in cell quota from the first to the second 3-d period in low P treatments. Thus growth rates were related to the cell quota before the period of growth, as long as external phosphorus concentrations were not very low. Use of as much as 600 pg per cell of internal phosphorus stores during the day 3–6 period likely fueled the high growth rates of *Microcystis* in low P treatments. Use of internal phosphorus stores during the day 0–3 period in high P treatments probably supported the higher growth rates in high P treatments during the day 0–3 versus the day 3–6 period.

The different timing of internal phosphorus use in low and high P treatments may be due to complex relationships with external phosphorus availability. According to simple internal phosphorus stores models, internal phosphorus should be available for algal growth and thereby result in the asymptotic relationship between growth rates and cell quota predicted by the Droop model. During the first 3-day period, *Microcystis* in low P treatments used internal phosphorus much less than in high P treatments. Recent research with marine phytoplankton shows that cells in oligotrophic conditions hoard phosphorus as polyphosphate and have completely different physiological responses in very low and high phosphorus conditions that result in uptake and persistent storage of polyphosphate in low phosphorus conditions [43–45]. Thus, *Microcystis* in our low P treatments conserved internal phosphorus stores until later in the experiment when conditions were more favorable for growth, whereas internal phosphorus stores were used relatively quickly in intermediate and high P treatments. This may be due to a tendency of algae in low phosphorus environments to conserve internal phosphorus for survival or until sufficient phosphorus is available for more rapid growth.

Increasing phosphorus concentrations in low P treatments due to cellular leakage of phosphorus from day 0 to 3 may have been the trigger for cells to use internal phosphorus stores in low P treatments from day 3 to 6. Phosphate concentration in low P treatments averaged 5 μg L^-1^, even in the lowest P treatments on day 3 and day 6. This increase in phosphorus concentration from the initial concentration of the culture media to measured concentrations on day 3 and day 6 is most likely due to leakage of phosphorus from *Microcystis* cells. Leakage of nutrients by phytoplankton has long been noted [46], and would be expected to be greatest when external phosphorus concentrations are low and the difference between concentrations inside and outside the cell are greatest. Leakage in our low P treatments seems reasonable when the mass of phosphorus in cells is related to the mass of phosphorus in our culture media. We conservatively assume 717 fg phosphorus in each cell at the beginning of the experiment, because that was the cell quota in the 256 P treatment on days 3 and 6. Since phosphorus concentrations in pre-culture conditions were higher than 111–116 μg L^-1^ averages of phosphorus concentrations in the high P treatment on days 3 and 6, cell quotas could be greater at the beginning of the experiment than our 717 fg cell^-1^ assumption. Leakage of 23% of phosphorus from 30,000 cells mL^-1^ in a culture with 717 fg cell^-1^ would increase nutrient concentrations in the low P cultures by 5 μg L^-1^.

The increase in phosphorus concentrations from less than 1 to 5 μg L^-1^ in low P treatments from day 0 to day 3 was probably sufficient to stimulate cells to use internal phosphorus stores for cell growth. Cell quota decreased with P treatment on day 3 between the 0 and 2 P treatments, indicating phosphorus concentrations in 2 P treatments were sufficient for cells to use internal phosphorus stores in growth. The half saturation constant estimated in the Monod Model for day 0–3 was 5.5 μg L^-1^. Thus, leakage of sufficient cellular phosphorus from day 0 to day 3 in the lowest P treatments was probably sufficient to increase external concentrations in the culture medium and to stimulate cells to use internal phosphorus stores for cell growth from day 3 to day 6. This use of 600 fg P cell^-1^ in low P treatments from day 3–6 could have fueled the higher growth rates in low P treatments than high P treatments during that period.

Cell demand for nutrients was not satisfied by the highest nutrient concentrations in the experiments. We did not observe an asymptotic relationship between phosphorus uptake rates and cell growth as predicted by the Michaelis-Menten Model [20]. Brown and Harris [47] showed inorganic phosphorus uptake rates do not saturate when cell quota was low. The cell quota in low and high P treatments were likely well below levels in pre-experiment culture conditions. Therefore, uptake rates may only saturate at phosphorus concentrations greater than 125 μg L^-1^ when phosphorus uptake exceeds potential use in growth and accumulation of internal phosphorus can saturate.

In conclusion, cell growth and phosphorus metabolism in non-equilibrium conditions is highly dynamic and varies greatly with phosphorus concentrations. We observed complex non-linear relationships that changed over time between external phosphorus concentrations, intracellular phosphorus stores, and cell growth. Simple Monod, Droop, and Michaelis-Menten models only partly explained growth and nutrient uptake rates during our 6-day experiment. These results could have great significance for understanding *Microcystis* growth in natural settings and for modeling response of *Microcystis* to phosphorus load and concentration management. Phosphorus hoarding in low phosphorus waters should be considered when using N:P and C:P ratios to understand nutrient limitation and when modeling algal growth in low phosphorus environments. The principles of Monod and Droop models were not wrong, they were just not complete. Both intracellular and external nutrients need to be considered when modeling algal growth, and changes in intracellular P rather than absolute concentrations were best related to growth in non-steady state conditions. Future experimental work on algal cell growth and nutrient metabolism with non-steady state conditions that simulate changes occurring in field settings will be particularly valuable for understanding and managing harmful algal blooms.

## Supporting information

S1 Dataset Data used for underlying figures and tables. (XLSX)
